# Development and validation of a survival nomogram and calculator for male patients with metastatic castration-resistant prostate cancer treated with abiraterone acetate and/or enzalutamide

**DOI:** 10.1186/s12885-023-10700-0

**Published:** 2023-03-07

**Authors:** Takashi Kawahara, Yusuke Saigusa, Shuko Yoneyama, Masashi Kato, Ippei Kojima, Hiroshi Yamada, Osamu Kamihira, Kenichi Tabata, Hideyasu Tsumura, Masatsugu Iwamura, Kazuhide Makiyama, Hiroji Uemura, Yasuhide Miyoshi

**Affiliations:** 1 Departments of Urology and Renal Transplantation, Yokohama City University Medical Center, 4-57 Urafune-Cho, Minami-Ku, Yokohama, 2320024 Japan; 2 Department of Biostatistics, Yokohama City University Graduate School of Medicine, Yokohama, 2360004 Japan; 3 Department of Urology, Nagoya University, Nagoya, 4668560 Japan; 4 Department of Urology, Kitasato University School of Medicine, Sagamihara, 2520375 Japan; 5 Department of Urology, Yokohama City University Graduate School of Medicine, Yokohama, 2320036 Japan

**Keywords:** CRPC, Prognosis, Enzalutamide, Abiraterone acetate, Nomogram

## Abstract

**Background:**

Despite the widespread availability of medication choices for metastatic castration-resistant prostate cancer (mCRPC), biomarkers to predict the efficacy of each mCRPC treatment have not yet been established. This study developed a prognostic nomogram and a calculator to predict the prognosis of patients with mCRPC who received abiraterone acetate (ABI) and/or enzalutamide (ENZ).

**Methods:**

In total, 568 patients with mCRPC who underwent ABI and/or ENZ between 2012 and 2017 were enrolled. A prognostic nomogram based on the risk factors was developed using the Cox proportional hazards regression model and clinically important factors. The discriminatory ability of the nomogram was assessed according to the concordance index (C-index). A 5-fold cross-validation was repeated 2000 times to estimate the C-index, and the means of the estimated C-index for the training and validation sets were determined. A calculator based on this nomogram was then developed.

**Results:**

The median overall survival (OS) was 24.7 months. Multivariate analysis showed that the time to CRPC, pre-chemotherapy, baseline prostate-specific antigen, baseline alkaline phosphatase, and baseline lactate dehydrogenase levels were independent risk factors for OS (hazard ratio [HR]: 0.521, 1.681, 1.439, 1.827, and 12.123, *p* = 0.001, 0.001,  < 0.001, 0.019, and  < 0.001, respectively). The C-index was 0.72 in the training cohort and 0.71 in the validation cohort.

**Conclusions:**

We developed a nomogram and calculator to predict OS in Japanese patients with mCRPC who received ABI and/or ENZ. Reproducible prognostic prediction calculators for mCRPC will facilitate greater accessibility for clinical use.

**Supplementary Information:**

The online version contains supplementary material available at 10.1186/s12885-023-10700-0.

## Background

A recent clinical trial revealed the efficacy of abiraterone acetate (ABI), enzalutamide (ENZ), Radium-223 (Ra-223), and cabazitaxel in addition to docetaxel chemotherapy for metastatic castration-resistant prostate cancer (mCRPC) patients [1–3]. Poly (ADP-ribose) polymerase (PARP) and immune checkpoint inhibitors are expected to be used clinically in the coming years for similar purposes [4, 5]. With several medication choices now available, clinicians should take care to select the most appropriate medicine to provide the optimal treatment. Prognostic prediction is important because of the lack of biomarkers for predicting the efficacy of individual mCRPC treatment. Recent studies have demonstrated the efficacy of tumor markers, such as inflammatory markers (e.g., neutrophil-to-lymphocyte, monocyte-to-lymphocyte, and platelet-to-lymphocyte ratios, as well as alkaline phosphatase (ALP) and lactate dehydrogenase (LDH) levels) for predicting prognosis [6].

However, accurate prognostic prediction requires a nomogram involving multiple prognostic parameters [7, 8]. Using clinical information, the present study developed and validated a nomogram for predicting the prognosis of male patients with mCRPC who received ABI and/or ENZ treatment and developed an Excel-based prognostic calculator for clinical use.

## Methods

### Study aim, design, and setting

#### Patients

A total of 568 mCRPC patients who received ABI and/or ENZ at Yokohama City University, Nagoya University, Kitasato University, and affiliated hospitals between 2012 and 2017 were evaluated. All patients were pathologically confirmed to have prostate cancer and received androgen deprivation therapy (ADT) but had refractory disease. CRPC was defined according to the Prostate Cancer Working Group 2 guidelines [9]. The patient characteristics, including initial prostate-specific antigen (PSA) level, initial metastatic status, Gleason score, observation period, time to CRPC, chemotherapy-naïve or not, age at baseline, PSA level at baseline, ALP level at baseline, LDH level at baseline, and initial ABI/ENZ treatment, are obtained from electronic medical records.

This study was approved by the Institutional Review Board of the Yokohama City University Medical Center (D1603004) and was conducted according to the tenets of the Declaration of Helsinki.

#### Treatment protocol

The choice for ENZ or ABI administration was decided by the urologist. In Japan, new hormonal therapies, such as ABI and/or ENZ, are generally used as the first-line therapies for mCRPC. Docetaxel followed by cabazitaxel is administered if the patients are eligible for chemotherapy. Ra-223 is also used in patients with bone-metastatic mCRPC without visceral or bulky lymph node metastases. Oral steroids or palliative radiotherapy is provided for terminally ill patients. No patients treated with PARP inhibitors were included in this study.

#### Variable definition

The chemotherapy status was defined according to whether or not docetaxel chemotherapy for CRPC was administered before initiating ABI/ENZ. The ALP levels were measured using a kit developed by the Japan Society of Clinical Chemistry (JSCC). In this study, ALP values in the nomogram and Excel-based calculator were calculated based on the International Federation of Clinical Chemistry and Laboratory Medicine (IFCC) guidelines more widely used worldwide. The ALP (IFCC) values were calculated using the approximate formula described by Hata et al. [10]: ALP (IFCC) value = ALP (JSCC) value × 0.337 + 2.959.

#### Nomogram development

A nomogram for overall survival (OS) was developed using a Cox proportional hazards regression model with age, initial PSA level, initial stage (M0/M1), Gleason score, time to CRPC, chemotherapy, PSA level at baseline, ALP level at baseline, and LDH level at baseline as predictors. Some of these factors were not independent prognostic variables in the multivariate analysis. However, they are established to be clinically important factors and were thus incorporated into the nomogram owing to their significant role in treatment decision making for CRPC.

Calibration was performed using the method described by Iasonos et al. [11]. The data were randomly separated into training and validation datasets to calibrate the nomogram prediction. The predictive ability was evaluated by comparing the predicted survival probability at 2 years with the observed survival probability, using the training and validation datasets. The discriminatory ability of the nomogram was assessed according to the concordance index (C-index). A 5-fold cross-validation was repeated 2000 times to estimate the C-index, and the mean values of the estimated C-index were determined using the training and validation sets. The decision curves obtained using the method described by Vickers et al. [12] for the 1- and 2-year survival rates predicted using the multivariable Cox model.

### Statistical analyses

OS was calculated from the date of baseline evaluation (initial ABI/ENZ treatment data) to the last follow-up. OS rates were estimated using the Kaplan–Meier method. A Cox proportional hazards model was used for univariate and multivariate analyses. The PSA, LDH, and ALP levels were used as continuous values. All statistical analyses were performed using SPSS (version 25.0; SPSS Inc., Chicago, IL, USA), R version 3.5.1 (R Foundation for Statistical Computing, Vienna, Austria), and GraphPad Prism (La Jolla, CA, USA). Statistical significance was set at *P* < 0.05.

## Results

The median (range) observational period was 13.3 (0.2–52.2) months. A total of 189 of the 568 patients (33.3%) died, and the median OS was 24.7 months (Supplementary Fig. 1). There was no difference in OS between patients treated first with ABI and those treated first with ENZ (Supplementary Fig. 2). A total of 202 patients (35.6%) were administered docetaxel systemic chemotherapy for CRPC and developed treatment resistance. The patients received docetaxel chemotherapy before ENZ/ABI initiation. None of the patients received either upfront ABI or ENZ during the initial treatment of metastatic hormone-naïve prostate cancer. Overall, 13 of the 568 (2.3%) patients were administered ABI or ENZ in the clinical trials. Patient characteristics are summarized in Table 1. The multivariate analysis showed that the time to CRPC (hazard ratio [HR] = 0.521, 95% confidence interval [CI] = 0.349–0.776, *p* = 0.001), chemotherapy naïve or not (HR = 1.681, 95% CI = 1.232–2.300, *p* = 0.001), baseline PSA level (HR = 1.439, 95% CI = 1.179–1.755, *p* < 0.001), baseline ALP level (HR = 1.827, 95% CI = 1.102–3.028, *p* = 0.019), and baseline LDH level (HR = 12.123, 95% CI = 5.343–27.51, *p* < 0.001) were independent risk factors for OS (Table 2).

**Table 1:** Patient characteristics

Variables	Total population	Treatment with ABI first	Treatment with ENZ first
Initial PSA level, ng/mL	123.0 [2.1–19840.0]	127.5 [2.4–14900.0]	122.5 [2.1–19840.0]
Initial stage			
M0	215 (37.9%)	97 (17.1%)	118 (20.8%)
M1	353 (62.1%)	137 (24.1%)	216 (38.0%)
Gleason score			
6–7	128 (22.5%)	60 (10.6%)	68 (12.0%)
8–10	440 (77.5%)	174 (30.6%)	266 (46.8%)
Observation period, months	13.3 [0.2–52.2]	12.9 [0.5–52.2]	13.4 [0.2–32.1]
Time to CRPC, months	14.2 [0.4–189.1]	15.0 [0.9–143.0]	14.0 [0.4–189.1]
Previous use of chemotherapy	202 (35.6%)	62 (10.9%)	140 (24.6%)
Age at baseline, years	76 [47–92]	76 [47–91]	76 [51–92]
PSA level at baseline, ng/mL	23.6 [0.1–10000.0]	20.8 [0.1–9500.0]	16.5 [0.1–10000.0]
ALP level at baseline, IU/L	261 [60–6908]	263 [60–6908]	261 [84–5421]
LDH level at baseline, IU/L	215 [93–3201]	215 [97–1628]	214 [93–3201]
Treatment			
ABI	234 (41.2%)	-	-
ENZ	334 (58.8%)	-	-

Data are presented as the n (%) or as the median [range]

*PSA* prostate-specific antigen, *ALP* alkaline phosphatase, *LDH* lactate dehydrogenase, *CRPC* castration-resistant prostate cancer, *ABI* abiraterone acetate, *ENZ* enzalutamide

**Table 2:** Univariate and multivariate analyses of influencing factors of prognosis

	Univariable analysis				Multivariable analysis			
	*p* value	HR	95% CI		*p* value	HR	95% CI	
			Lower	Upper			Lower	Upper
Initial PSA level	0.820	1.020	0.863	1.204	0.157	0.862	0.701	1.059
Initial stage (M0 vs M1)	0.091	0.776	0.578	1.042	0.469	0.876	0.611	1.254
Gleason score (8–10 vs 6–7)	0.848	1.034	0.737	1.450	0.550	0.897	0.629	1.280
Time to CRPC	< 0.001	0.434	0.304	0.621	0.001	0.521	0.349	0.776
Chemotherapy (yes vs no)	< 0.001	2.300	1.731	3.057	0.001	1.683	1.232	2.300
Age at baseline (years)	0.600	0.995	0.975	1.015	0.250	1.012	0.992	1.032
PSA level at baseline	< 0.001	1.958	1.680	2.282	< 0.001	1.439	1.179	1.755
ALP level at baseline	< 0.001	5.138	3.509	7.524	0.019	1.827	1.102	3.028
LDH level at baseline	< 0.001	45.886	23.15	90.953	< 0.001	12.123	5.343	27.51

*PSA* prostate-specific antigen, *ALP* alkaline phosphatase, *LDH* lactate dehydrogenase, *CRPC* castration-resistant prostate cancer, *HR* hazard ratio, *CI* confidence interval

**Fig. 1:**
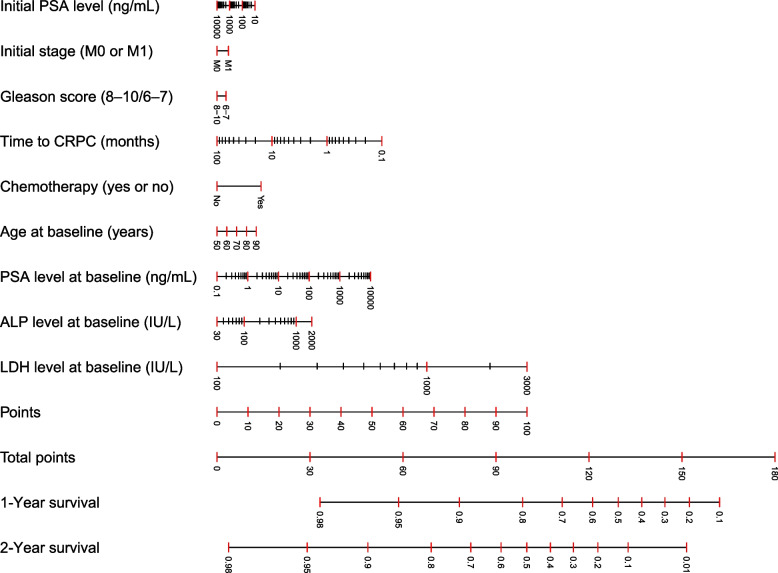
Nomogram for predicting 1- and 2-year survival in mCRPC treated with abiraterone acetate and/or enzalutamide

**Fig. 2:**
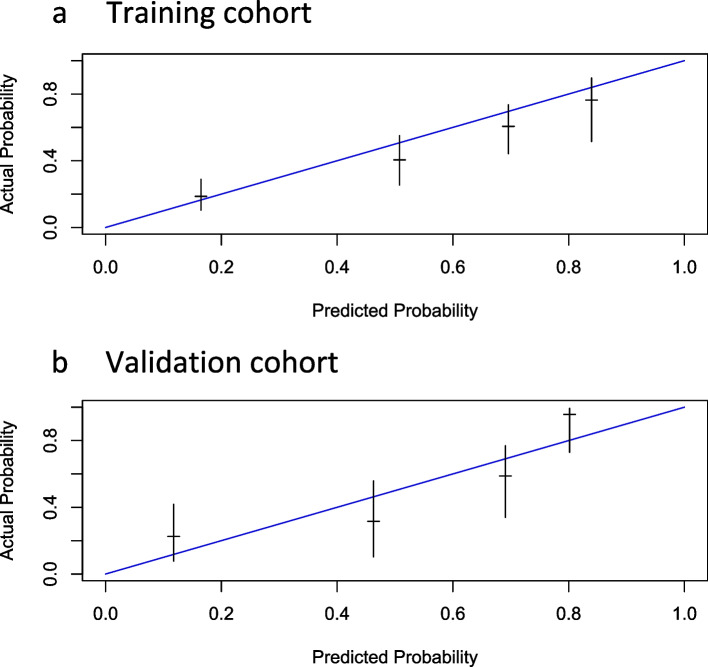
Calibration plots of the nomogram for predicting 2-year survival. **a** Training cohort. **b** Validation cohort. The blue diagonal line indicates the ideal reference line, at which the probabilities match the observed proportions. The vertical lines across the blue line represent the nomogram-predicted probabilities grouped for each of the four quartile groups along with the respective 95% confidence intervals

**Fig. 3:**
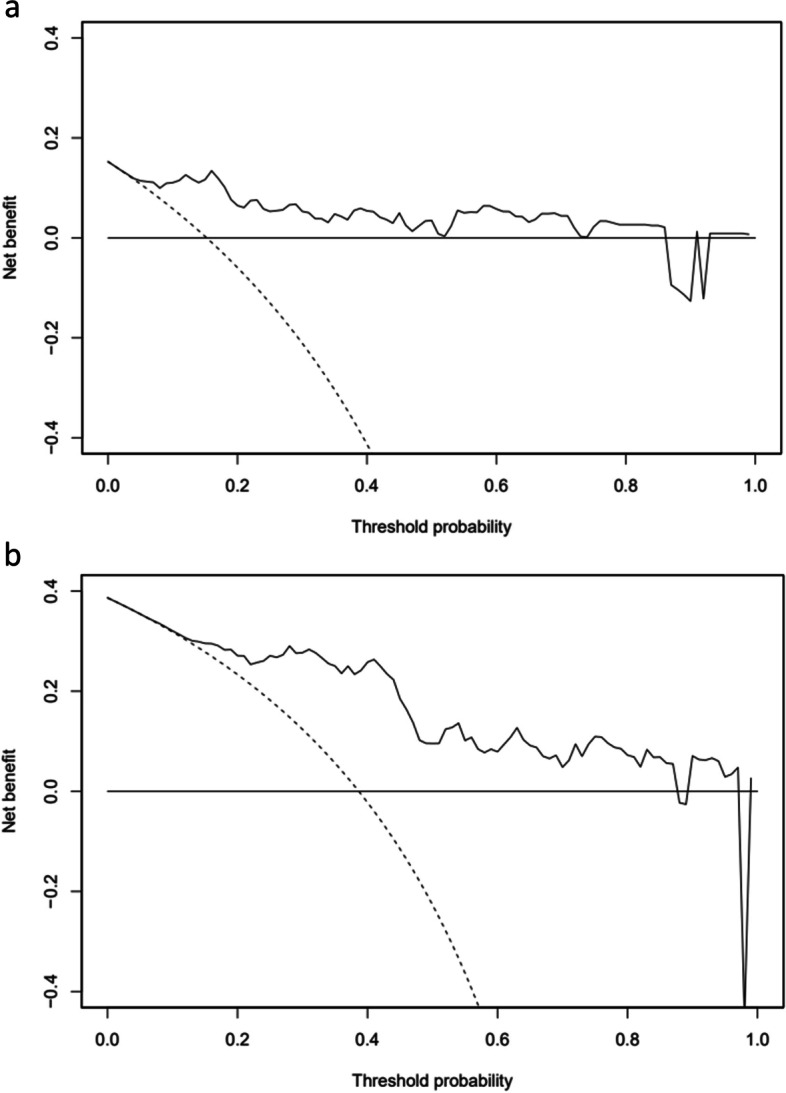
Decision curve for survival obtained using the multivariable Cox model. **a** The 1-year survival. **b** The 2-year survival. The dashed curve line is the net benefit of treating all patients; the black curve line is the net benefit of treating high-risk patients based on the prediction model; the black horizontal line is the net benefit of treating no patient

Figure 1 shows the nomogram for predicting 1- and 2-year survival using five statically significant risk factors (i.e., time to CRPC, chemotherapy-naïve or not, baseline PSA, baseline ALP, and baseline LDH) and four clinically important risk factors (i.e., age, initial PSA, initial metastatic status, and Gleason score). Figure 2a and b show the calibration of the nomogram for predicting the 2-year survival. As shown in Fig. 2a, the predicted 2-year survival rate from the nomogram correlated well with the actual 2-year survival rate in the training dataset. Figure 2b shows the calibration of the nomogram for predicting the 2-year survival using a randomly selected validation dataset. The 2-year survival rate predicted by the nomogram correlated well with the actual 2-year survival rate in the validation set. The mean C-index was 0.72 for the training cohort and 0.71 for the validation cohort in the 5-fold cross-validation. Figure 3a and b show the decision curves for the 1- and 2-year survival rates predicted using the multivariable Cox model. We also developed an Excel-based calculator for convenient application of these findings in daily clinical practice (Supplementary File 1).

## Discussion

This study developed and validated a nomogram to predict the prognosis of patients with mCRPC who received ABI and/or ENZ. The nomogram was composed of the initial PSA; initial metastasis status; Gleason score; time to CRPC; previous use of docetaxel; age at ABI/ENZ initiation; and laboratory data, including PSA, ALP, LDH levels at the time of ABI/ENZ installation.

The prognosis of mCRPC varies according to the location of metastatic lesions. For non-metastatic CRPC (m0CRPC), the PROSPER, SPARTAN, and ARAMIS studies showed a median radiographic progression-free survival of 36.6–40.4 months [13–15]. Although the final median OS is not reached, it is expected to be approximately 67.0–73.9 months [16]. Regarding chemotherapy-naïve mCRPC, the PREVAIL and COU-AA-302 studies reported an OS of 32.4–34.7 months [1, 17]. For mCRPC after docetaxel chemotherapy, the AFFIRM and COUA-AA-301 reported an OS of 14.8–18.4 months [2, 18]. In the real world, the OS for mCRPC patients is 31.6 months for those with lymph node metastasis, 21.3 months for those with bone metastasis, 19.4 months for those with lung metastasis, and 13.5 months for those with liver metastasis [19]. Although OS has been speculated to differ according to the metastatic site, a detailed prognostic prediction model involving multiple risk factors has not been established. Timely and appropriate treatment decision making for the right patient requires a detailed prognostic estimation.

Previous studies have reported prognostic factors for metastatic prostate cancer. With respect to tumor markers, systemic inflammatory markers including the neutrophil-to-lymphocyte ratio, monocyte-to-lymphocyte ratio, lymphocyte-to-platelet ratio, and De Ritis ratio, and the prognostic nutritional index have all been identified to be prognostic factors for prostate cancer [6, 20, 21]. In addition, geriatric and sarcopenia statuses have been reported as new prognostic factors for CRPC [22]. Recent studies have shown that elevated levels of tumor markers (e.g., LDH and ALP phosphatase) and of some inflammatory markers are poor prognostic factors for CRPC [6]. Yang et al. developed a nomogram using the presence of liver metastasis, hemoglobin levels, and time from initial ADT to ABI treatment in 110 Chinese patients [23]. Lin et al. developed a nomogram using PSA doubling time, time to PSA progression, and the presence of pain in 167 Chinese patients [24]. For patients who underwent ABI, Khalaf et al. and Chi et al. developed nomograms using data from 197 and 762 patients, respectively. Consistent with these findings, the current study found that high LDH levels and a short response to luteinizing hormone-releasing hormone were poor prognostic factors. However, these studies included mostly non-Asian cohorts [25, 26]. The survival rate of patients with prostate cancer in Asian countries is higher than that of patients in Western countries. Further, prostate cancer prognosis differs across countries within Asia [27]. Therefore, a nomogram comprising data from a single country is important for accurate survival predictions.

Similar to other studies, we created nomograms to predict OS in 568 mCRPC patients who received ABI/ENZ. We were able to collect important information from both patients and physicians. Although various nomograms have been reported, the current nomogram is advantageous in that it includes a prognostic calculator that can be used in daily clinical practice.

The present study has several limitations. First, the study used a retrospective cohort from multiple Japanese hospitals, and the patients were divided into the training and control groups. Most hospitals are tertiary referral cancer centers. Thus,an external validation study is required to confirm the accuracy and applicability of the findings in other hospitals, including private clinics. Second, this study did not include any upfront treatment for metastatic hormone-naïve prostate cancer (mHNPC). In Japan, upfront docetaxel, apalutamide, and ENZ have been approved for mHNPC by the Japanese National Insurance System based on the findings of the CHAARTD, TITAN, and ARCHES studies. These drugs were approved in addition to ABI for high-risk mHNPC, which was approved based on the results of the LATITUDE trial. Further studies are required to confirm the efficacy of the nomogram in patients receiving upfront treatment for mHNPC.

Third, our nomogram did not include other influencing factors of prognosis such as the ECOG-PS, hemoglobin levels, or metastatic location, all of which strong predicted survival in men with mCRPC [23, 28]. However, our database did not include information on ECOG-PS or metastatic location. Finally, the observational period was short. Most patients were followed up for less than 30 months. In addition, 198 (34.9%) patients died during the follow-up period, and no correlation was observed between the covariates and the observational period.

## Conclusions

We developed and validated a nomogram for predicting the prognosis of Japanese patients with mCRPC who received ABI/ENZ treatment and an Excel-based prognostic calculator for use in clinical practice. The nomogram showed excellent predictive ability and may thus help clinicians make treatment decisions for patients with mCRPC.

## Supplementary Information

**Additional file 1:** **Supplementary Fig. 1.** Kaplan–Meier curve of overall survival in metastatic castration-resistant prostate cancer patients treated with abiraterone and/or enzalutamide.

**Additional file 2:** **Supplementary Fig. 2.** Kaplan–Meier curve of overall survival in metastatic castration-resistant prostate cancer patients treated with abiraterone first or enzalutamide first.

**Additional file 3:** **Supplementary file 1.**

## Data Availability

Data presented in this study are available from the corresponding author on reasonable request. However, these data are not available publicly.

## Abbreviations

mCRPC: Metastatic castration-resistant prostate cancer
ABI: Abiraterone acetate
ENZ: Enzalutamide
OS: Overall survival
C-index: Concordance index
PSA: Prostate-specific antigen
ALP: Alkaline phosphatase
LDH: Lactate dehydrogenase
HR: Hazard ratio
CI: Confidence interval
Ra-223: Radium-223
PARP: Poly (ADP-ribose) polymerase
ADT: Androgen deprivation therapy
JSCC: Japan Society of Clinical Chemistry
IFCC: International Federation of Clinical Chemistry and Laboratory Medicine
mHNPC: Metastatic hormone-naïve prostate cancer
