# Supplementary material for: Development and validation of a survival nomogram and calculator for male patients with metastatic castration-resistant prostate cancer treated with abiraterone acetate and/or enzalutamide
Source: BMC Cancer. 2023 Mar 7;23:214. doi: 10.1186/s12885-023-10700-0 (PMC9990312; doi:10.1186/s12885-023-10700-0)
Supplement: Supplementary file 1 — Additional file 1: Supplementary Fig. 1. Kaplan–Meier curve of overall survival in metastatic castration-resistant prostate cancer patients treated with abiraterone and/or enzalutamide. [file 12885_2023_10700_MOESM1_ESM.pptx]

## Slide 1
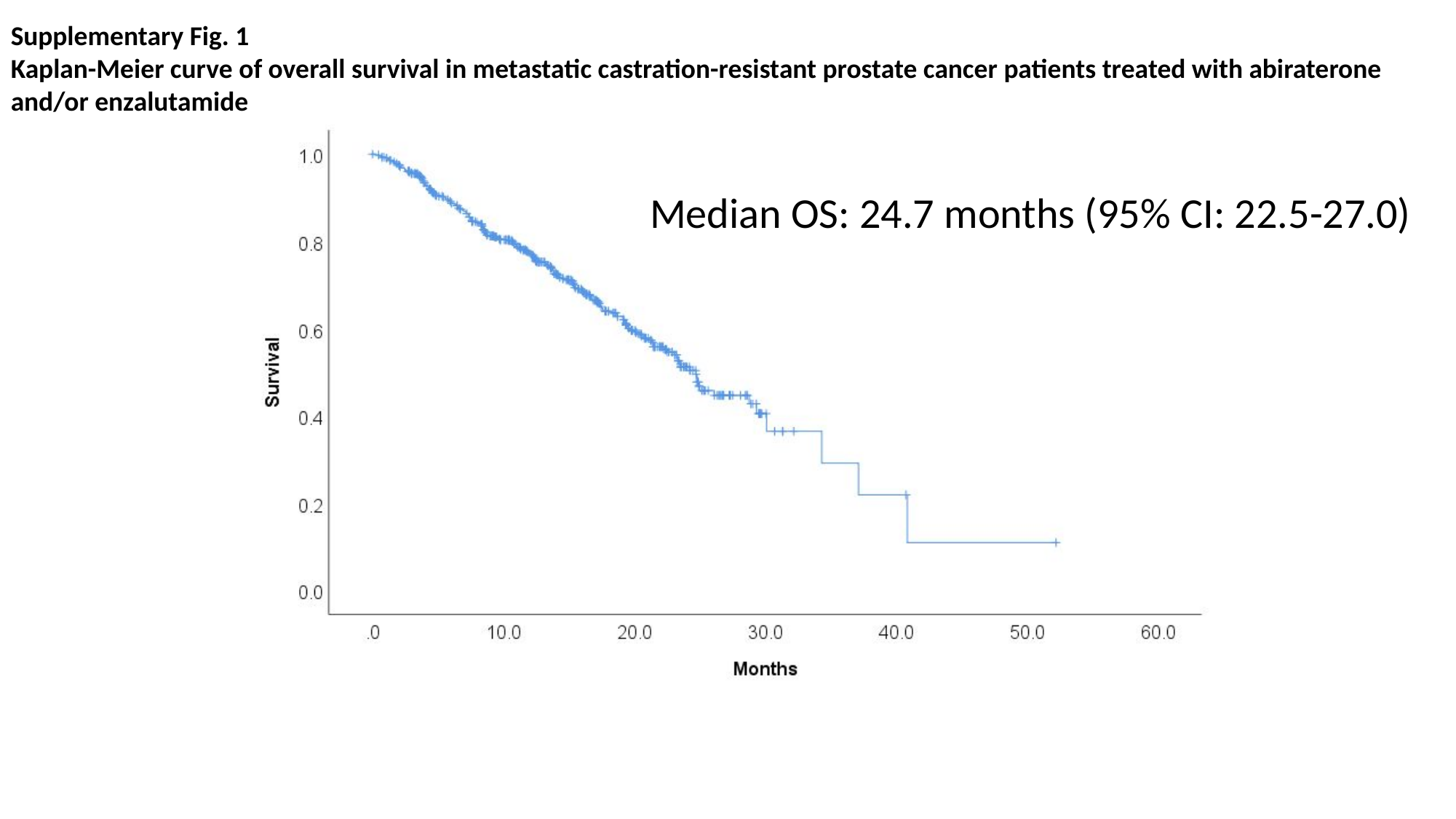

Supplementary Fig. 1
Kaplan-Meier curve of overall survival in metastatic castration-resistant prostate cancer patients treated with abiraterone and/or enzalutamide
Median OS: 24.7 months (95% CI: 22.5-27.0)
