# Supplementary material for: Development and validation of a survival nomogram and calculator for male patients with metastatic castration-resistant prostate cancer treated with abiraterone acetate and/or enzalutamide
Source: BMC Cancer. 2023 Mar 7;23:214. doi: 10.1186/s12885-023-10700-0 (PMC9990312; doi:10.1186/s12885-023-10700-0)
Supplement: Supplementary file 2 — Additional file 2: Supplementary Fig. 2. Kaplan–Meier curve of overall survival in metastatic castration-resistant prostate cancer patients treated with abiraterone first or enzalutamide first. [file 12885_2023_10700_MOESM2_ESM.pptx]

## Slide 1
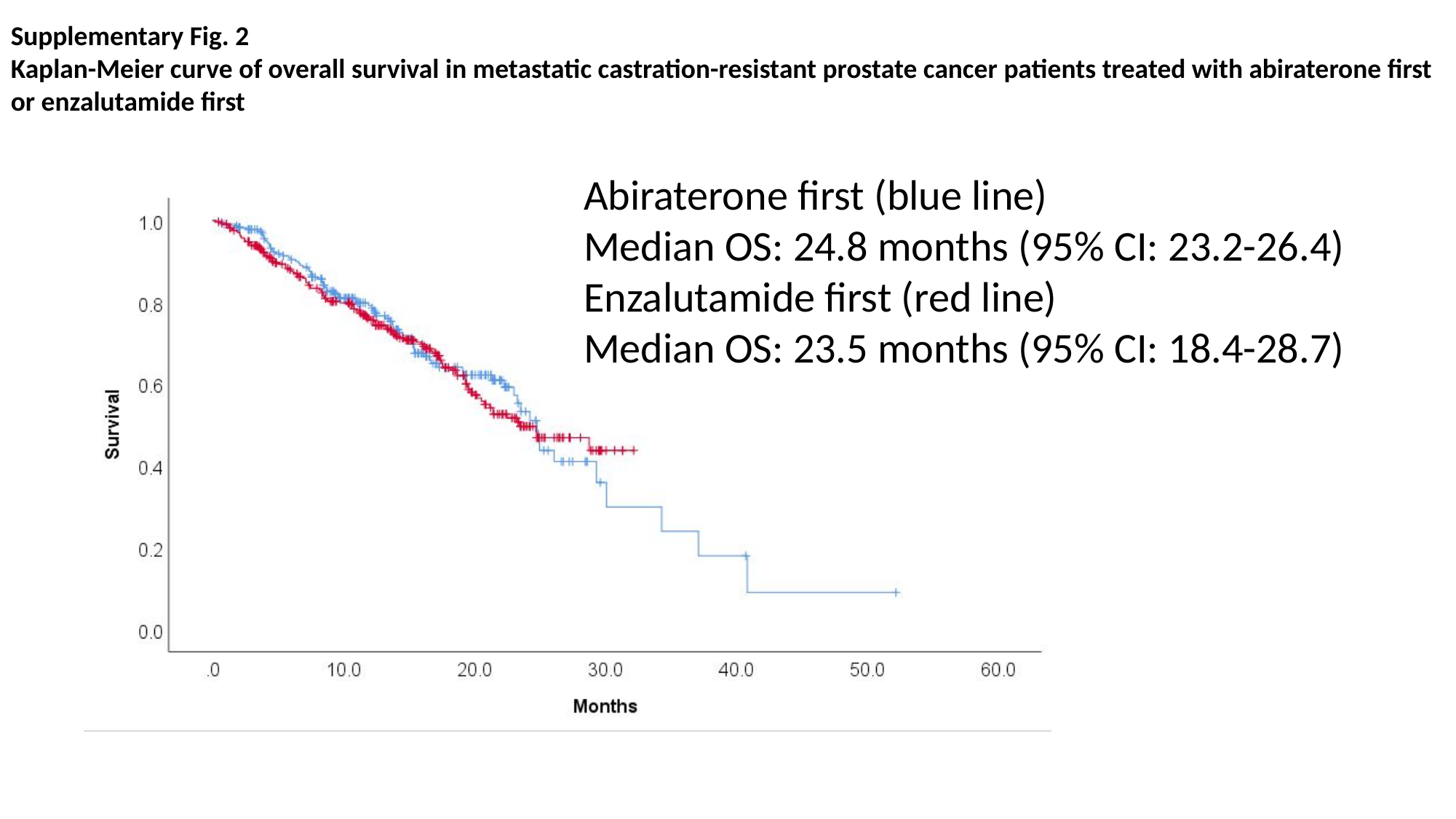

Supplementary Fig. 2
Kaplan-Meier curve of overall survival in metastatic castration-resistant prostate cancer patients treated with abiraterone first or enzalutamide first
Abiraterone first (blue line)
Median OS: 24.8 months (95% CI: 23.2-26.4)
Enzalutamide first (red line)
Median OS: 23.5 months (95% CI: 18.4-28.7)
